# Fatty infiltration of periarticular muscles in patients with osteonecrosis of the femoral head

**DOI:** 10.1007/s00264-025-06457-9

**Published:** 2025-02-20

**Authors:** Keiji Otaka, Yusuke Osawa, Yasuhiko Takegami, Hiroki Iida, Hiroto Funahashi, Shiro Imagama

**Affiliations:** 1 Nagoya University, Nagoya, Japan; 2 Nagoya Central Hospital, Nagoya, Japan

**Keywords:** Fatty infiltration, Muscle, Osteonecrosis of the femoral head

## Abstract

**Purpose:**

Muscle mass and fatty infiltration can be assessed on computed tomography (CT) images using the cross-sectional area (CSA) and computed tomography attenuation value (CTV). Femoral head collapse in osteonecrosis of the femoral head (ONFH) may affect both values. We investigated factors influencing the CSA and CTV of the periarticular muscles in patients with ONFH.

**Methods:**

Overall, 101 patients with ONFH with unilateral hip pain (stage 2, 24 patients; stage 3 A, 49 patients; and stage 3B, 28 patients) were included. The CSA and mean CTV of the bilateral gluteus maximus (Gmax), gluteus medius (Gmed), gluteus minimus (Gmin), and iliopsoas (IP) muscles were measured using CT cross-sections. Bilateral comparisons and associations with Japanese Investigation Committee (JIC) stage were analysed. Multiple regression analysis was used to evaluate factors associated with the CSA and CTV.

**Results:**

On the symptomatic side, the CSA was significantly lower for the Gmax, Gmed, and IP, whereas the CTV was significantly lower for all tested muscles (all *p* < 0.01). The CTV, but not the CSA, of the Gmax, Gmed, and Gmin was significantly associated with the JIC stage severity bilaterally (all *p* < 0.01). Multiple regression analysis showed significant associations of the CTV with age, sex, and JIC stage (all *p* < 0.01).

**Conclusion:**

Symptomatic ONFH leads to decreased muscle mass and increased fatty infiltration. Femoral head collapse progression is associated with a decrease in the CTV. Periarticular muscle assessment, including on the contralateral side, is important in patients with ONFH, particularly in older women.

**Supplementary Information:**

The online version contains supplementary material available at 10.1007/s00264-025-06457-9.

## Introduction

Osteonecrosis of the femoral head (ONFH) is a progressive hip disorder that predominantly affects young and middle-aged adults and leads to femoral head collapse and joint destruction. Collapse results in severe pain and reduced activities of daily living (ADL) [1–3]. Reduced activity levels can induce muscle mass reduction and fatty infiltration [4]. In hip disorders, muscle mass reduction and fatty infiltration of the periarticular muscles can occur on the affected side [5]. Considering the worsening of pain and decreased activity levels associated with ONFH [6–8], it is reasonable to postulate that similar changes would occur in the periarticular muscles of patients with ONFH. However, studies evaluating the periarticular muscles in patients with ONFH are lacking.

Muscle mass reduction can be assessed by measuring the cross-sectional area (CSA) of the muscle by using computed tomography (CT) or magnetic resonance imaging, while fatty infiltration can be evaluated by measuring the CT attenuation value (CTV), expressed in Hounsfield units (HU) [9, 10]. In patients with ONFH, pain-related disuse and altered weight-bearing patterns may manifest as reductions in the CSA and CTV [11]. As the collapse progresses and activity levels decline [7], muscle mass reduction and fatty infiltration may concomitantly worsen. The CSA and CTV have been reported as predictors of outcomes after total hip arthroplasty (THA) [12–14], emphasizing their importance in hip disorders. Furthermore, the condition of the muscle of the contralateral limbs is crucial. Recent studies have highlighted the significance of contralateral lower limb status in post-THA physical function [15, 16]. Consequently, changes in muscle mass and quality on the asymptomatic side are non-negligible factors in postoperative function and rehabilitation strategies.

We hypothesized that the CSA and CTV of the periarticular muscles in patients with ONFH would be associated with the progression of femoral head collapse, not only on the symptomatic side, but also on the asymptomatic side. This study evaluated the CSA and CTV of the gluteus maximus (Gmax), gluteus medius (Gmed), gluteus minimus (Gmin), and iliopsoas (IP) muscles in patients with ONFH, and assessed their correlation with the Japanese Investigation Committee (JIC) stage classification. Additionally, we identified factors that influenced the CSA and CTV of the gluteus medius.

In this study, we addressed the following questions: (1) Do patients with ONFH exhibit reduced CSA and CTV in the periarticular muscles on the symptomatic side as compared to those on the asymptomatic side? (2) Are the CSA and CTV on the symptomatic side associated with JIC stage classification severity? (3) Are the CSA and CTV on the asymptomatic side associated with JIC stage classification severity? (4) What risk factors are associated with a decrease in the CSA and CTV in the periarticular muscles?

## Materials and methods

### Patients

This study involved a retrospective chart review and was approved by our institutional review board. Between January 2019 and July 2024, 196 patients with ONFH were admitted to our hospital. Of these, patients with traumatic necrosis (*n* = 12), missing data (*n* = 25), a history of hip surgery (*n* = 5), and JIC classification stage 4 (*n* = 8) were excluded, as were 45 patients with bilateral hip pain symptoms. Finally, 101 patients with pain symptoms on only one side and no symptoms in the contralateral hip were included. The patient demographics are presented in Table 1. More than half were men. The mean age was 40.8 years (range 13–83 years). Forty-four patients had bilateral ONFH. The aetiology of ONFH was most often steroid-associated, followed by alcohol-associated, and unknown causes. The stage and type of ONFH were classified according to the JIC criteria [17]. Most were in stage 3 A (collapse of the femoral head < 3 mm), followed by stage 2 (sclerosis without collapse of the femoral head), and then stage 3B (collapse of the femoral head ≥ 3 mm). None of the patients had stage 1 ONFH. No significant differences were observed in age, height, weight, body mass index (BMI), or aetiology among the stages. Stage 2 had significantly fewer type B cases and stage 3B had a significantly longer duration since onset. Stage severity was associated with the Japanese Orthopaedic Association (JOA) range-of-motion (ROM) score. Sex-specific results are shown in Supplementary Tables 1 and 2.

**Table 1:** Patient demographics by JIC stage classification

	Total	Stage 2	Stage 3 A	Stage 3B	*P* value
Number of patients	101	24	49	28	
Age, years (SD)	40.8 (14.0)	41.0 (13.8)	41.3 (14.4)	39.5 (14.0)	0.910*
Sex, n (%)					0.827†
Men	63 (62.4)	14 (58.3)	32 (65.3)	17 (60.7)	
Women	38 (37.6)	10 (41.7)	17 (34.7)	11 (39.3)	
Height, cm (SD)	165.2 (8.1)	166.7 (8.6)	164.9 (8.5)	164.5 (7.3)	0.428*
Body weight, kg (SD)	63.1 (13.1)	63.8 (10.4)	63.8 (14.1)	61.5 (13.8)	0.690*
BMI, kg/m^2^ (SD)	23.5 (4.0)	22.9 (3.2)	23.4 (4.5)	22.6 (4.0)	0.788*
Bilateral ONFH, n (%)	44 (43.6)	9 (37.5)	23 (46.9)	12 (42.9)	0.744†
Duration from onset, months (SD)	6.1 (8.6)	2.8 (1.8)	5.5 (6.3)	10.3 (13.0)	< 0.001*§
Associated risk factors, n					0.129†
Steroid	50	11	28	11	
Alcohol	44	9	20	15	
Idiopathic	7	4	1	2	
JIC type classification, n					< 0.001†¶
B	3	3	0	0	
C1	38	16	16	6	
C2	60	5	33	22	
JOA hip score of symptomatic side (SD)					
Pain	20.3 (9.6)	23.5 (8.9)	19.4 (9.3)	18.9 (10.6)	0.062‡
ROM	16.2 (3.4)	17.7 (3.4)	16.2 (3.1)	14.8 (3.3)	< 0.001‡
Gait	13.0 (4.7)	14.3 (4.6)	12.2 (4.5)	13.3 (4.9)	0.270‡
ADL	14.0 (3.6)	14.6 (3.8)	13.3 (3.8)	14.7 (3.0)	0.601‡
Total	63.7 (17.9)	70.1 (17.4)	61.1 (17.5)	62.5 (18.1)	0.037‡

* Kruskal–Wallis test

† Chi-squared test

‡ Jonckheere–Terpstra trend test

§ Stage 2 compared with Stage 3B.

¶ Stage 2 compared with Stage 3 A and 3B.

JIC, Japanese Investigation Committee; SD, standard deviation; BMI, body mass index; ONFH, osteonecrosis of the femoral head; JOA, Japanese Orthopaedic Association; ROM, range-of-motion; ADL, activity of daily life

## CT imaging and measurements

**Fig. 1:**
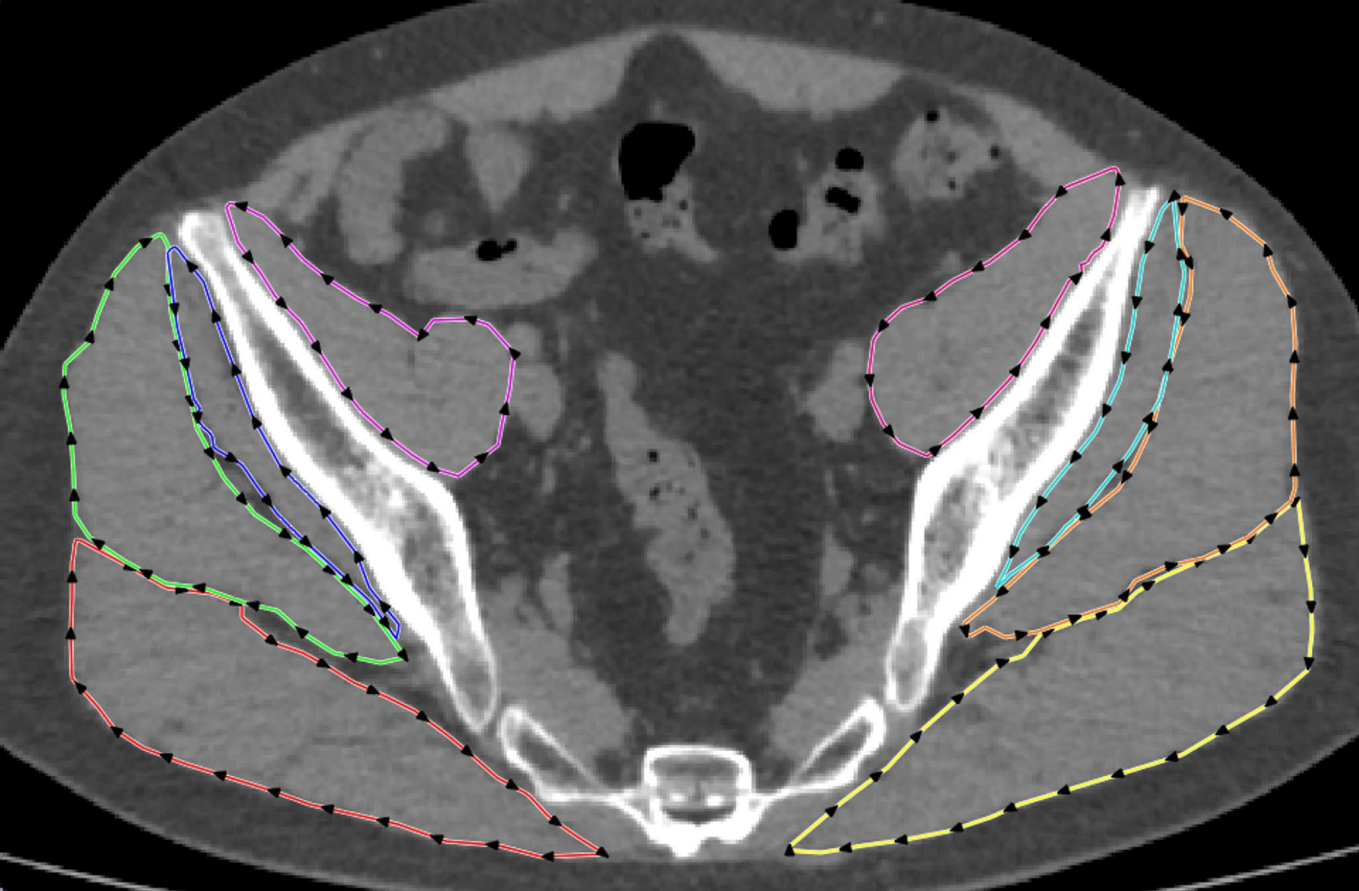
Computed tomography (CT) axial image of a 43-year-old male with right ONFH Stage 3 A. The boundaries of the bilateral gluteus maximus, gluteus medius, gluteus minimus, and iliopsoas muscles were manually plotted. The cross-sectional areas (CSA) and mean CT attenuation values (CTV) were automatically calculated using analysis software

Periarticular muscle was evaluated using CT (Aquilion One; Toshiba Medical Systems Co., Tochigi, Japan). Briefly, the patients were placed in a supine position, and CT images were acquired with a 1-mm slice-thickness. Data were stored in Digital Imaging and Communications in Medicine format, and the CSA and mean CTV of the Gmax, Gmed, Gmin, and IP of muscles were measured at the horizontal cross-sectional level, immediately distal to the sacroiliac joint [18]. Measurements were performed using SliceOmatic image analysis software (version 5.0; TomoVision, Montreal, QC, Canada). As previously reported, muscle fascia was manually plotted, and the CSA and mean CTV were automatically measured by the analysis software (Fig. 1) [19]. Since the total muscle mass is related to body weight [20], the CSA was normalized to body weight (mm2/kg) and expressed as the N-CSA. To assess reliability, two physicians (K. O. and Y. O.) evaluated 10 samples, twice. The intra-observer and inter-observer intraclass correlation coefficients were 0.92 and 0.90 for the CSA, and 0.92 and 0.89 for the CTV, respectively.

## Data analyses

The painful side was defined as the symptomatic side, and the pain-free side as the asymptomatic side. The N-CSA and CTV were compared between sides using paired t-tests. For comparison of the three groups defined by JIC stage classification, statistical significance of differences in continuous variables between groups were analysed using one-way analysis of variance or the Kruskal–Wallis test, depending on the data distribution, and categorical variables were analysed using the chi-square test. The Jonckheere–Terpstra trend test was used to assess the association between the JIC stage classification severity and the JOA scores, N-CSA, and CTV. Factors influencing the CSA and CTV of the Gmed on the symptomatic side were evaluated using multiple regression analysis. All statistical analyses were performed using EZR software program (Saitama Medical Center, Jichi Medical University) [21]. *P* < 0.05 was considered statistically significant.

## Results

Table 2 shows a comparison between the symptomatic and asymptomatic hips of all patients. The N-CSA was significantly lower on the symptomatic side for the Gmax, Gmed, and IP, but no significant difference was observed for the Gmin. The CTV was significantly lower on the symptomatic side for all muscles. Sex-specific results are presented in Supplementary Table 3.

**Table 2:** Comparison of N-CSA and CTV between symptomatic and asymptomatic sides

	Symptomatic side	Asymptomatic side	*P* value*
N-CSA, mm^2^/kg (SD)			
Gmax	45.4 (9.1)	49.9 (9.1)	< 0.001
Gmed	41.3 (8.1)	45.3 (7.7)	< 0.001
Gmin	13.7 (4.0)	13.5 (4.0)	0.497
IP	21.0 (4.6)	23.2 (5.2)	< 0.001
CTV, HU (SD)			
Gmax	31.7 (12.8)	35.8 (10.9)	< 0.001
Gmed	43.2 (9.4)	46.9 (6.6)	< 0.001
Gmin	35.7 (15.5)	41.0 (12.5)	< 0.001
IP	54.2 (7.6)	55.8 (6.4)	0.002

* paired *t*-test

N-CSA, normalized cross-sectional area; CTV, computed tomography attenuation value; SD, standard deviation; Gmax, gluteus maximus; Gmed, gluteus medius; Gmin, gluteus minimus; IP, iliopsoas; HU, Hounsfield unit

Table 3 shows the N-CSA and CTV for both symptomatic and asymptomatic sides at each stage. The CSA was not associated with the JIC stage on either side. The CTV significantly decreased with increasing JIC stage on both sides for the Gmax, Gmed, and Gmin. The IP showed an association only on the symptomatic side. Sex-specific results are presented in Supplementary Table 4.

**Table 3:** Association of JIC stage classification with N-CSA and CTV

	Symptomatic side					Asymptomatic side				
	Stage 2	Stage 3 A	Stage 3B	*P* value*		Stage 2	Stage 3 A	Stage 3B	*P* value*	
N-CSA, mm^2^/kg (SD)										
Gmax	47.1 (9.6)	45.8 (9.3)	43.0 (8.3)	0.083		49.2 (9.4)	51.1 (9.4)	48.6 (8.3)	0.408	
Gmed	42.5 (8.6)	41.2 (7.8)	40.6 (8.5)	0.300		44.8 (8.2)	45.6 (7.4)	45.2 (7.9)	0.583	
Gmin	13.6 (4.0)	13.5 (3.9)	14.0 (4.3)	0.647		13.5 (3.9)	13.5 (4.0)	13.4 (4.1)	0.337	
IP	22.4 (5.0)	20.8 (4.4)	20.3 (4.6)	0.060		23.2 (5.6)	22.7 (5.1)	23.6 (5.1)	0.756	
CTV, HU (SD)										
Gmax	38.9 (7.6)	30.6 (13.2)	28.9 (10.9)	< 0.001		41.8 (7.5)	34.8 (11.1)	32.3 (11.1)	< 0.001	
Gmed	49.1 (5.2)	42.8 (9.0)	38.9 (10.5)	< 0.001		50.4 (4.6)	46.3 (6.8)	44.7 (6.5)	< 0.001	
Gmin	43.6 (12.5)	35.8 (13.2)	28.6 (18.6)	< 0.001		46.1 (11.7)	40.5 (11.7)	37.5 (13.5)	0.004	
IP	57.6 (6.4)	54.1 (6.7)	51.5 (8.9)	0.006		57.3 (5.6)	55.6 (5.8)	55.0 (8.0)	0.250	

* Jonckheere–Terpstra trend test

JIC, Japanese Investigation Committee; N-CSA, normalized cross-sectional area; CTV, computed tomography attenuation value; SD, standard deviation; Gmax, gluteus maximus; Gmed, gluteus medius; Gmin, gluteus minimus; IP, iliopsoas; HU, Hounsfield unit

Multiple regression analysis revealed that being a woman, BMI, and JIC stage 3B were factors significantly influencing the decrease in the CSA of the Gmed. Additionally, being a woman, having advanced age, and JIC Stages 3 A and 3B were factors significantly influencing a decrease in the CTV of the Gmed (Table 4).

**Table 4:** Multiple regression analysis of factors associated with CSA and CTV of Gluteus medius on symptomatic side

	CSA				CTV		
	β	95%CI	*P* value		β	95%CI	*P* value
Age, years	−0.0001	−0.166 to 0.166	0.999		−0.431	−0.606 to − 0.257	< 0.001
Sex (women)	−1.033	−1.363 to − 0.702	< 0.001		−0.630	−0.977 to − 0.283	< 0.001
BMI, kg/m^2^	0.255	0.094 to 0.417	0.002		−0.106	−0.275 to 0.063	0.217
Duration from onset, months	0.007	−0.157 to 0.172	0.931		−0.051	−0.224 to 0.126	0.557
Associated risk factors							
Steroid	−0.186	−0.529 to 0.157	0.284		0.058	−0.301 to 0.418	0.748
Alcohol	Reference						
Idiopathic	−0.482	−1.129 to 0.166	0.143		0.269	−0.411 to 0.948	0.434
JIC Stage classification							
2	Reference						
3 A	−0.331	−0.731 to − 0.068	0.103		−0.665	−1.085 to − 0.246	0.002
3B	−0.481	−0.938 to − 0.025	0.039		−1.075	−1.554 to − 0.595	< 0.001

CSA, cross-sectional area; CTV, computed tomography attenuation value; CI, confidence interval; BMI, body mass index; JIC, Japanese Investigation Committee

## Discussion

This study revealed that the CSA of the Gmax, Gmed, and IP, as well as the CTV of the Gmax, Gmed, Gmin, and IP, were significantly lower on the symptomatic side than on the contralateral side in patients with ONFH. We found that the deterioration of the JIC stage was associated with a decrease in the CTV of the Gmax, Gmed, and Gmin on both the symptomatic and asymptomatic sides. Furthermore, advanced age and being a woman were identified as additional risk factors for CTV reduction in the Gmed. These findings suggested the need for an evaluation of periarticular muscles in patients with ONFH, particularly in older women with collapse of the femoral head. To our knowledge, no previous study has evaluated muscle mass and fatty infiltration of the periarticular muscles in patients with ONFH or demonstrated their association with the JIC stage classification of ONFH.

Hip pain leads to a reduction in muscle mass and fatty infiltration in the affected limb. The results of this study suggested that these changes are likely due to the disuse of the symptomatic hip caused by decreased activity and pain. Reduced physical activity has been reported to decrease muscle mass as well as increase intermuscular adipose tissue (IMAT). Manini et al. reported that subjects who underwent 4 weeks of unilateral limb suspension experienced a significant increase in IMAT and a decrease in muscle volume in the thigh and calf of the suspended limb as compared to the contralateral side [4]. Zacharias et al. found that patients with osteoarthritis exhibited lower activity levels and noted a decrease in muscle volume of the Gmax, Gmed, and Gmin, as well as increased levels of fatty infiltration in the Gmax and Gmin on the affected side [5]. Similar reductions in activity levels have been reported in patients with ONFH [2, 7], which likely contributed to the decreased muscle mass and increased fatty infiltration observed.

The CSA of muscle has been reported to be associated with body weight, whereas the CTV is related to age [20, 22, 23]. In this study, we normalized the CSA by body weight, and found no significant differences in age when the patients were grouped by JIC stage. Therefore, these factors were largely eliminated from the comparison among the three groups. Our study revealed that, as the severity of the JIC stage increased, the CTV of the Gmax, Gmed, and Gmin deteriorated on the symptomatic side. Kawano et al. reported that pain and the angle of hip abduction were associated with abductor torque [24]. Momose et al. reported that muscle volume and muscle CT density were factors associated with hip abductor strength [25]. In our study, the ROM of the JOA score was also associated with the JIC stage severity, and although not statistically significant, pain showed a similar trend. It is possible that the deterioration in pain and ROM led to worsening of the CTV. However, we did not find an association between the CSA and JIC stage. The change in the CSA may have been underestimated because it did not consider the influence of intramuscular fat tissue and noncontractile tissue [26, 27]. A decrease in the CTV has been reported to indicate a loss of contractile muscle [27], while CTV is more closely related to physical function than is the CSA [19, 28]. Therefore, evaluation of the CTV may be more important than that of the CSA. To maintain ADL, deterioration of the CTV must be prevented.

*Recent studies have reported that the functional state of the contralateral lower limb is associated with postoperative activity levels* [15, 16]. Hamada et al. revealed preoperative lower extremity muscle weakness on the contralateral side in patients with steroid-related ONFH as compared to patients with osteoarthritis [29]. These findings underscored the importance of evaluating the contralateral limb in ONFH patients.Our study revealed that the CTV of the Gmax, Gmed, and Gmin on the asymptomatic side was also associated with JIC stage classification. Previous reports have shown that the collapse of the femoral head is related to patients’ ADL and quality of life [7]. However, in our study, we did not find an association between gait and the ADL components of the JOA score. Therefore, the relationship between muscle atrophy and decreased activity levels was considered weak. ONFH has been reported to be a systemic inflammatory disease [30, 31]. Additionally, steroids and alcohol, which are major causes of ONFH, are known to induce muscle atrophy [32–34]. These systemic factors may have contributed to our findings. Based on the results of this study, appropriate therapeutic interventions, such as rehabilitation, including that of the asymptomatic side, should be implemented before the JIC stage progresses, in order to maintain the CTV.

This study revealed that the JIC stage and sex were associated with the CSA and CTV of the periarticular muscles in patients with ONFH, and that age was associated with the CTV. In the general population, the CSA and CTV have been reported to be lower in older individuals and in women [22], with sex hormones, adipose tissue mass, and intramyocellular lipid content thought to be contributing factors [35, 36]. However, we did not find significant differences in the aetiology of ONFH. This may be because both steroid use and alcohol consumption, which account for almost all causes of ONFH, are known to induce muscle atrophy [32–34]. THA is an important treatment option for ONFH in older patients, and muscle CSA and CTV have been reported to be important for postoperative physical function in hip surgery [12–14]. Other studies have reported that older individuals and women have worse postoperative physical function and improvement after THA [37, 38], which may be influenced by the muscle CSA and CTV. Muscle atrophy is reported as a risk factor of dislocation and persist for two years after THA[39, 40]. In cases of severe atrophy of the periarticular muscles, the use of dual-mobility bearings or large-diameter femoral heads should be considered to enhance joint stability and reduce the risk of dislocation.

This study had some limitations. First, the sample size was small. However, the incidence of ONFH is low and patients with pain on only one side are even rarer, making it difficult to recruit a large number of patients. Second, while muscle attenuation determined by CT was associated with muscle lipid content determined in muscle biopsy specimens[41], fatty infiltration in this study did not represent an actual increase in lipid content based on muscle biopsy. Third, this study did not consider the causes of ONFH or comorbidities that could affect the muscles’ condition. Steroids, alcohol, and diseases, such as malignancies, which necessitate steroid administration have been reported to cause muscle atrophy [32–34, 42]. To investigate these effects, future studies need to clarify the duration and amount of steroid and alcohol intake, as well as related diseases. Furthermore, the measurements of the muscle CSA and CTV depended on the location of the CT image slices. Finally, the CSA measurements may have underestimated changes in the CSA because noncontractile tissues were not excluded [26, 27]. Despite these limitations, the results of this study, which focused on muscle mass and fatty infiltration in patients with ONFH, provide valuable insights, and suggested the need for preoperative muscle analysis in the ONFH cases undergoing THA.

In conclusion, the periarticular muscles of patients with ONFH showed greater muscle mass reduction and more marked fatty infiltration on the symptomatic side than did those on the asymptomatic side. The CTV of the periarticular muscles was associated with the JIC stage classification of ONFH not only on the symptomatic side but also on the asymptomatic side. CTV was related to the JIC stage classification, age, and sex. It is important to assess the periarticular muscles, including those of the contralateral side, in patients with ONFH, and particularly in older women.

## Electronic supplementary material

Below is the link to the electronic supplementary material.

Supplementary Material 1

## Data Availability

No datasets were generated or analysed during the current study.
