# Supplementary material for: Fatty infiltration of periarticular muscles in patients with osteonecrosis of the femoral head
Source: Int Orthop. 2025 Feb 20;49(5):1073–80. doi: 10.1007/s00264-025-06457-9 (PMC12003464; doi:10.1007/s00264-025-06457-9)
Supplement: Supplementary file 1 — Supplementary Material 1 [file 264_2025_6457_MOESM1_ESM.docx]

| **Supplementary Table 1** Demographics of men by JIC stage classification. | | | | | |
| --- | --- | --- | --- | --- | --- |
|  | **Total** | **Stage 2** | **Stage 3A** | **Stage 3B** | ***P* value** |
| Number of patients | 63 | 14 | 32 | 17 |  |
| Age, years (SD) | 43.0 (11.7) | 43.1 (10.0) | 43.7 (13.2) | 41.6 (10.2) | 0.449* |
| Height, cm (SD) | 170.2 (5.2) | 172.6 (4.2) | 169.7 (5.8) | 169.2 (4.5) | 0.16† |
| Body weight, kg (SD) | 68.2 (12.4) | 67.3 (10.0) | 68.4 (13.7) | 68.7 (12.1) | 0.946* |
| BMI, kg/m^2^ (SD) | 23.5 (4.0) | 22.6 (3.2) | 23.7 (4.5) | 23.9 (3.8) | 0.617* |
| Bilateral ONFH, n (%) | 24 (38.1) | 4 (28.6) | 13 (40.6) | 7 (41.2) | 0.707‡ |
| Duration from onset, months (SD) | 5.7 (5.8) | 3.3 (2.1) | 6.0 (6.7) | 7.3 (5.6) | 0.051* |
| Associated risk factors, n |  |  |  |  | 0.171‡ |
| Steroid | 27 | 6 | 16 | 5 |  |
| Alcohol | 33 | 6 | 16 | 11 |  |
| Idiopathic | 3 | 2 | 0 | 1 |  |
| JIC type classification, n |  |  |  |  | 0.047‡¶, 0.024‡** |
| B | 0 | 0 | 0 | 0 |  |
| C1 | 22 | 10 | 9 | 3 |  |
| C2 | 41 | 4 | 23 | 14 |  |
| JOA hip score of symptomatic side (SD) |  |  |  |  |  |
| Pain | 22.0 (9.9) | 26.8 (6.7) | 22.3 (8.7) | 17.6 (12.4) | 0.015§ |
| ROM | 16.3 (2.9) | 17.9 (1.9) | 16.4 (2.6) | 14.7 (3.2) | <0.001§ |
| Gait | 13.9 (4.5) | 14.9 (4.7) | 13.3 (4.4) | 14.3 (4.6) | 0.413§ |
| ADL | 14.5 (3.4) | 15.3 (3.4) | 14.1 (3.6) | 14.6 (3.1) | 0.333§ |
| Total | 66.7 (17.3) | 74.9 (13.9) | 65.9 (16.6) | 61.2 (19.3) | 0.014§ |
| * Kruskal–Wallis test † One-way analysis of variance ‡ Chi-squared test § Jonckheere–Terpstra trend test ¶ Stage 2 compared with Stage 3A. ** Stage 2 compared with Stage 3B. JIC, Japanese Investigation Committee; SD, standard deviation; BMI, body mass index; ONFH, osteonecrosis of the femoral head; JOA, Japanese Orthopaedic Association; ROM, range of motion; ADL, activity of daily life | | | | | |

| **Supplementary Table 2** Demographics of women by JIC stage classification. | | | | | |
| --- | --- | --- | --- | --- | --- |
|  | **Total** | **Stage 2** | **Stage 3A** | **Stage 3B** | ***P* value** |
| Number of patients | 38 | 10 | 17 | 11 |  |
| Age, years (SD) | 37.1 (16.7) | 38.2 (18.1) | 37.0 (15.8) | 36.2 (18.4) | 0.933* |
| Height, cm (SD) | 157.1 (4.6) | 158.4 (5.6) | 156.2 (4.6) | 157.2 (3.6) | 0.325* |
| Body weight, kg (SD) | 54.8 (9.6) | 58.8 (9.3) | 55.3 (10.5) | 50.4 (7.1) | 0.136* |
| BMI, kg/m^2^ (SD) | 22.3 (4.1) | 23.4 (3.3) | 22.7 (4.7) | 20.5 (3.4) | 0.113* |
| Bilateral ONFH, n (%) | 20 (52.6) | 5 (50) | 10 (58.8) | 5 (45.5) | 0.772† |
| Duration from onset, months (SD) | 5.7 (5.8) | 2.0 (1.2) | 4.6 (5.6) | 14.8 (19.2) | 0.004*§ |
| Associated risk factors, n |  |  |  |  | 0.739† |
| Steroid | 23 | 5 | 12 | 6 |  |
| Alcohol | 11 | 3 | 4 | 4 |  |
| Idiopathic | 4 | 2 | 1 | 1 |  |
| JIC type classification, n |  |  |  |  | 0.020†¶, 0.018†§ |
| B | 3 | 3 | 0 | 0 |  |
| C1 | 16 | 6 | 7 | 3 |  |
| C2 | 19 | 1 | 10 | 8 |  |
| JOA hip score of symptomatic side (SD) |  |  |  |  |  |
| Pain | 17.9 (9.3) | 19.0 (9.9) | 14.1 (8.0) | 22.7 (9.0) | 0.780‡ |
| ROM | 16.0 (4.1) | 17.3 (5.0) | 15.8 (3.9) | 15.0 (3.5) | 0.020‡ |
| Gait | 11.6 (4.7) | 13.6 (4.7) | 10.4 (4.2) | 11.8 (5.1) | 0.208‡ |
| ADL | 13.3 (3.9) | 13.6 (4.3) | 12.0 (3.9) | 14.9 (3.1) | 0.792‡ |
| Total | 58.8 (17.9) | 63.5 (20.2) | 52.3 (16.1) | 64.5 (16.7) | 0.478‡ |
| * Kruskal–Wallis test † Chi-squared test ‡ Jonckheere–Terpstra trend test § Stage 2 compared with Stage 3B. ¶ Stage 2 compared with Stage 3A. JIC, Japanese Investigation Committee; SD, standard deviation; BMI, body mass index; ONFH, osteonecrosis of the femoral head; JOA, Japanese Orthopaedic Association; ROM, range of motion; ADL, activity of daily life | | | | | |

| **Supplementary Table 3** Comparison of N-CSA and CTV between symptomatic and asymptomatic sides by sex. | | | |
| --- | --- | --- | --- |
|  | **Symptomatic side** | **Asymptomatic side** | ***P* value***** |
| **Men** |  |  |  |
| N-CSA, mm^2^/kg (SD) |  |  |  |
| Gmax | 47.5 (8.5) | 52.5 (8.4) | <0.001 |
| Gmed | 42.0 (8.3) | 46.6 (7.8) | <0.001 |
| Gmin | 14.8 (3.7) | 14.5 (3.2) | 0.385 |
| IP | 21.9 (4.7) | 24.3 (5.0) | <0.001 |
| CTV, HU (SD) |  |  |  |
| Gmax | 34.2 (11.3) | 38.4 (9.1) | <0.001 |
| Gmed | 44.6 (8.9) | 48.7 (5.4) | <0.001 |
| Gmin | 40.3 (12.7) | 45.2 (10.3) | <0.001 |
| IP | 55.9 (6.5) | 57.3 (5.3) | 0.007 |
| **Women** |  |  |  |
| N-CSA, mm^2^/kg (SD) |  |  |  |
| Gmax | 41.7 (9.1) | 45.7 (8.6) | 0.001 |
| Gmed | 40.2 (7.9) | 43.1 (7.1) | 0.002 |
| Gmin | 11.7 (3.9) | 11.7 (4.5) | 0.998 |
| IP | 19.7 (4.1) | 21.3 (5.1) | <0.001 |
| CTV, HU (SD) |  |  |  |
| Gmax | 27.5 (14.1) | 31.2 (12.3) | <0.001 |
| Gmed | 40.9 (9.8) | 43.8 (7.1) | 0.002 |
| Gmin | 28.0 (16.9) | 33.7 (13.5) | 0.002 |
| IP | 51.3 (8.4) | 53.4 (7.4) | 0.063 |
| * paired *t*-test N-CSA, normalized cross-sectional area; CTV, computed tomography attenuation value; SD, standard deviation; Gmax, gluteus maximus; Gmed, gluteus medius; Gmin, gluteus minimus; IP, iliopsoas; HU, Hounsfield unit | | | |

| **Supplementary Table 4** Association of JIC stage classification with N-CSA and CTV by sex. | | | | | | | | | | |
| --- | --- | --- | --- | --- | --- | --- | --- | --- | --- | --- |
|  | **Symptomatic side** | | | |  | **Asymptomatic side** | | | | |
|  | **Stage 2** | **Stage 3A** | **Stage 3B** | ***P* value*** |  | **Stage 2** | **Stage 3A** | **Stage 3B** | ***P* value*** |  |
| **Men** |  |  |  |  |  |  |  |  |  |  |
| N-CSA, mm^2^/kg (SD) |  |  |  |  |  |  |  |  |  |  |
| Gmax | 50.1 (9.6) | 48.1 (7.7) | 44.6 (8.2) | 0.030 |  | 53.2 (9.8) | 53.4 (7.7) | 50.5 (8.7) | 0.109 |  |
| Gmed | 44.2 (8.2) | 41.8 (8.3) | 40.8 (8.5) | 0.199 |  | 46.9 (6.5) | 47.1 (7.8) | 45.4 (9.1) | 0.255 |  |
| Gmin | 14.7 (3.7) | 14.5 (3.8) | 15.6 (3.4) | 0.812 |  | 14.9 (3.5) | 14.7 (3.1) | 13.9 (3.1) | 0.188 |  |
| IP | 23.9 (5.3) | 21.7 (4.5) | 20.6 (4.5) | 0.027 |  | 24.4 (6.0) | 24.0 (5.0) | 24.7 (4.2) | 0.715 |  |
| CTV, HU (SD) |  |  |  |  |  |  |  |  |  |  |
| Gmax | 40.6 (7.5) | 34.2 (11.7) | 28.9 (10.9) | 0.001 |  | 43.8 (6.9) | 38.3 (9.8) | 34.5 (6.9) | <0.001 |  |
| Gmed | 49.8 (4.4) | 44.6 (9.0) | 40.3 (9.6) | <0.001 |  | 51.5 (3.4) | 49.0 (5.4) | 45.9 (5.7) | 0.001 |  |
| Gmin | 47.4 (5.9) | 40.9 (11.4) | 33.2 (15.7) | <0.001 |  | 50.6 (4.7) | 45.2 (8.8) | 40.7 (11.9) | 0.008 |  |
| IP | 59.6 (5.6) | 55.8 (6.0) | 53.0 (6.7) | 0.003 |  | 58.3 (6.2) | 57.1 (5.3) | 56.9 (4.6) | 0.214 |  |
| **Women** |  |  |  |  |  |  |  |  |  |  |
| N-CSA, mm^2^/kg (SD) |  |  |  |  |  |  |  |  |  |  |
| Gmax | 42.9 (8.2) | 41.7 (10.6) | 40.6 (8.2) | 0.314 |  | 43.6 (5.2) | 46.8 (10.9) | 45.8 (7.2) | 0.877 |  |
| Gmed | 40.1 (9.1) | 40.3 (6.9) | 40.3 (8.8) | 0.543 |  | 41.7 (9.7) | 42.8 (5.8) | 44.9 (6.2) | 0.938 |  |
| Gmin | 12.0 (4.2) | 11.7 (3.5) | 11.4 (4.5) | 0.286 |  | 11.5 (3.7) | 11.2 (4.6) | 12.7 (5.4) | 0.656 |  |
| IP | 20.4 (4.1) | 19.0 (3.8) | 20.0 (4.8) | 0.394 |  | 21.7 (4.7) | 20.8 (4.8) | 21.9 (6.2) | 0.564 |  |
| CTV, HU (SD) |  |  |  |  |  |  |  |  |  |  |
| Gmax | 36.6 (7.6) | 23.8 (13.4) | 24.8 (16.8) | 0.021 |  | 39.0 (7.9) | 28.2 (10.5) | 28.9 (15.6) | 0.058 |  |
| Gmed | 48.0 (6.3) | 39.3 (8.1) | 36.9 (12.0) | 0.004 |  | 49.0 (5.8) | 41.4 (6.5) | 42.9 (7.3) | 0.024 |  |
| Gmin | 38.3 (17.2) | 26.1 (10.7) | 21.6 (21.3) | 0.036 |  | 39.9 (15.6) | 31.5 (11.3) | 31.6 (14.2) | 0.089 |  |
| IP | 54.8 (6.3) | 50.8 (6.7) | 49.1 (11.6) | 0.089 |  | 56.0 (4.4) | 52.7 (5.6) | 52.1 (11.2) | 0.277 |  |
| * Jonckheere–Terpstra trend test JIC, Japanese Investigation Committee; N-CSA, normalized cross-sectional area; CTV, computed tomography attenuation value; SD, standard deviation; Gmax, gluteus maximus; Gmed, gluteus medius; Gmin, gluteus minimus; IP, iliopsoas; HU, Hounsfield unit | | | | | | | | | | |
